# Supplementary material for: Empirical data drift detection experiments on real-world medical imaging data
Source: Nat Commun. 2024 Feb 29;15:1887. doi: 10.1038/s41467-024-46142-w (PMC10904813; doi:10.1038/s41467-024-46142-w)
Supplement: Supplementary file 1 — Supplementary Information [file 41467_2024_46142_MOESM1_ESM.pdf]

Supplementary Information

Supplementary Tables

Metadata label	category	Proportion (%)	Metadata label	category	Proportion (%)
**view**	AP	79	**institution**	Institution #1	48
	PA	21		Institution #2	52
**sex**	M	58	**modality**	CR	57
	F	42		DX	43
**Patient age**	65+	62	**device**	Device #1	19
	35-65	32		Device #2	18
	18-35	6		Device #3	16
**Patient class**	Inpatient	57		Device #4	13
	Emergency	24		Device #5	13
	Outpatient	19		Device #6	9
**ICU Status**	False	78		Device #7	6
	True	22		Device #8	4

Supplementary Table 1. Detailed description of the dataset used for this study. For each meta-data tag, we provide the proportion of images corresponding to the tag.

	Value	Patient Age: 18-35	Institution #1	Institution #2	Patient class: Emergency	Patient class: Outpatient	Patient Class: Inpatient
Atelectasis	POSITIVE	0.06	-	-	-	-	-
Lung Opacity	POSITIVE	0.08	-	-	0.06	-	-
Pleural Effusion	POSITIVE	0.07	-	-	0.06	-	-
Support Devices	POSITIVE	0.08	0.06	-	0.12	-	-
device	Device #1	-	-	0.09	-	-	-
	Device #4	-	-	0.08	-	-	-
	Device #3	0.05	0.08	-	-	-	-
	Device #2	0.05	0.09	-	-	-	-
	Device #5	-	0.07	-	-	-	-
institution	Institution #1	-	-	-	-	-	0.05
	Institution #2	0.13	-	-	0.1	-	-
ICU status	False	-	-	-	-	-	0.08
	True	-	-	-	0.1	0.1	-
modality	CR	-	-	0.23	-	-	-
	DX	0.13	0.23	-	0.11	-	-
patient_age	65+	-	-	-	0.08	-	-
patient_class	Inpatient	0.16	0.06	-	-	-	-
view	AP	0.14	0.07	-	0.14	-	-
	PA	-	-	0.08	-	-	0.06

Supplementary Table 2. Tolerance of the final sample (0.5) for the experiments on patient metadata that rise above 5%. The columns in the table are each of the experiments and the rows are the categories that are being monitored. The tolerance for the experimental category for each experiment is omitted due to it being the value we purposely want to skew. For example, in the experiments where young patients (Age 18-35) were oversampled to be 50% of the target dataset (column 1), the proportion of cases of Atelectasis changed by 6% (row 1) when compared to the proportion of cases of Atelectasis in the source dataset, and the proportion of patients from Institution #2 changed by 13% (row 11). We tracked the deviation in proportions from the source dataset because these deviations could play a factor in drift detection (i.e., it's theoretically possible that the algorithm noticed the change in atelectasis instead of the intended synthetic drift of younger patients). We report these deviations over 5% for the target dataset where the synthetically enriched category is enriched to 50% because it represents the highest degree of variation experienced.

	Value	Atelectasis: 1	Consolidation: 1	Pleural Effusion: 1	Pneumonia: 1	Cardiomegaly: 1	Lung Lesion: 1	Edema: 1	Fracture: 1	Lung Opacity: 1	Enlarged Cardiomediastinum: 1
**Enlarged Cardiomediastinum**	**NEGATIVE**	-	-	0.07	-	0.07	-	0.06	-	-	-
**No Finding**	**POSITIVE**	0.13	0.12	0.13	0.13	0.12	0.12	0.13	0.13	0.12	0.13
**Support Devices**	**POSITIVE**	-	-	-	0.09	-	0.08	-	0.12	-	-
**device**	**Device #3**	-	-	-	-	-	-	-	0.06	-	-
	**Device #2**	-	-	-	-	-	-	-	0.05	-	-
**institution**	**Institution #2**	-	-	-	0.08	-	0.10	-	0.15	-	-
**ICU Status**	**True**	-	-	-	-	-	0.05	-	0.06	-	-
**modality**	**DX**	-	-	-	0.08	-	0.10	-	0.15	-	-
**patient_age**	**35-65**	-	-	-	-	-	-	0.07	-	-	-
**patient_class**	**Emergency**	-	-	0.05	-	-	-	-	-	-	-
	**Inpatient**	-	-	-	-	-	-	-	0.07	-	-
**view**	**AP**	-	-	-	-	-	0.06	-	-	-	-

Supplementary Table 3. Tolerance of the final sample (0.5) for the experiments on pathologies that rise above 5%. The columns in the table are each of the experiments and the rows are the categories that are being monitored. The tolerance for the experimental category for each experiment is omitted due to it being the value we purposely want to skew. For example, in the experiments where images with atelectasis were oversampled to be 50% of the target dataset (column 1), the proportion of cases of "No Findings" changed by 13% (row 2) when compared to the proportion of images with "No Finding" in the source dataset. We tracked the deviation in proportions from the source dataset because these

deviations could play a factor in the ability of test approaches to detect data drift. We tracked the deviation in proportions from the source dataset because these deviations could play a factor in drift detection (i.e., it’s theoretically possible that the algorithm noticed the change in No Findings instead of the intended synthetic drift of increased Atelectasis).

	Cardiomegaly	Consolidation	Edema	Effusion	Enlarged Cardiomediastinum	Fracture	Lung Lesion	Lung Opacity	Pleural Other	Pneumonia	Pneumothorax
AUROC	0.87	0.93	0.93	0.84	0.67	0.82	0.84	0.84	0.92	0.88	0.84
F1	0.63	0.62	0.66	0.44	0.16	0.20	0.38	0.61	0.37	0.47	0.26
Precision	0.75	0.67	0.77	0.74	0.53	0.56	0.26	0.84	0.72	0.68	0.68
Recall	0.55	0.57	0.59	0.32	0.10	0.12	0.70	0.48	0.25	0.35	0.16

Supplementary Table 4. Aggregate model metrics (AUROC, F1, precision, recall) broken down over all pathologies.

Supplementary Figures

In this appendix, we present the results of the drift experiments of TAE+BBSD and TAE with other performance macro-averaged measures (F1, Precision, and Recall).
We plot:

- **AUROC + Sub-populations: Supplementary Figure 1.** Synthetic categorical shift experiments of the TAE, TAE+BBSD, BBSD, and AUROC monitoring methods for shift in patient sub-populations. p-values were calculated using a two-sided multivariate maximum mean discrepancy (MMD) statistical test with no correction for multiple comparison. Data are presented as mean values +/- 1 standard deviation.
- **AUROC + Patient Pathologies: Supplementary Figure 2.** Synthetic categorical shift experiments of the TAE, TAE+BBSD, BBSD, and AUROC monitoring methods for shift in patient pathologies. p-values were calculated using a two-sided multivariate maximum mean discrepancy (MMD) statistical test with no correction for multiple comparison. Data are presented as mean values +/- 1 standard deviation.
- **F1 + Sub-populations: Supplementary Figure 3.** Synthetic categorical shift experiments of the TAE, TAE+BBSD, and F1 monitoring methods for shift in patient sub-populations. p-values were calculated using a two-sided multivariate maximum mean discrepancy (MMD) statistical test with no correction for multiple comparison. Data are presented as mean values +/- 1 standard deviation.
- **F1 + Patient Pathologies: Supplementary Figure 4.** Synthetic categorical shift experiments of the TAE, TAE+BBSD, and F1 monitoring methods for shift in patient pathologies. p-values were calculated using a two-sided multivariate maximum mean discrepancy (MMD) statistical test with no correction for multiple comparison. Data are presented as mean values +/- 1 standard deviation.
- **Recall + Sub-populations: Supplementary Figure 5.** Synthetic categorical shift experiments of the TAE, TAE+BBSD, and Recall monitoring methods for shift in patient sub-populations. p-values were calculated using a two-sided multivariate maximum mean discrepancy (MMD) statistical test with no correction for multiple comparison. Data are presented as mean values +/- 1 standard deviation.
- **Recall + Patient Pathologies: Supplementary Figure 6.** Synthetic categorical shift experiments of the TAE, TAE+BBSD, and Recall monitoring methods for shift in patient pathologies. p-values were calculated using a two-sided multivariate maximum mean discrepancy (MMD) statistical test with no correction for multiple comparison. Data are presented as mean values +/- 1 standard deviation.
- **Precision + Sub-populations: Supplementary Figure 7.** Synthetic categorical shift experiments of the TAE, TAE+BBSD, and Precision monitoring methods for shift in patient sub-populations. p-values were calculated using a two-sided multivariate maximum mean discrepancy (MMD) statistical test with no correction for multiple comparison. Data are presented as mean values +/- 1 standard deviation.
- **Precision + Patient Pathologies: Supplementary Figure 8.** Synthetic categorical shift experiments of the TAE, TAE+BBSD, and Precision monitoring methods for shift in patient pathologies. p-values were calculated using a two-sided multivariate maximum mean discrepancy (MMD) statistical test with no correction for multiple comparison. Data are presented as mean values +/- 1 standard deviation.
- **Brier Score + Sub-populations: Supplementary Figure 9.** Synthetic categorical shift experiments of the TAE, TAE+BBSD, and Brier Score monitoring methods for shift in patient sub-populations. p-values were calculated using a two-sided

multivariate maximum mean discrepancy (MMD) statistical test with no correction for multiple comparison. Data are presented as mean values +/- 1 standard deviation.

- **Brier Score + Patient Pathologies: Supplementary Figure 10.** Synthetic categorical shift experiments of the TAE, TAE+BBSD, and Brier Score monitoring methods for shift in patient pathologies. p-values were calculated using a two-sided multivariate maximum mean discrepancy (MMD) statistical test with no correction for multiple comparison. Data are presented as mean values +/- 1 standard deviation.
- **Precision + Patient Pathologies: Supplementary Figure 11.** Data drift as detected by TAE+BBSD (image-and-output-based) drift detection over time on real-world data overlaid with performance monitoring broken down over pathologies. p-values were calculated using a two-sided multivariate maximum mean discrepancy (MMD) statistical test with no correction for multiple comparison. Data are presented as mean values +/- 1 standard deviation.
- **Precision + Patient Pathologies: Supplementary Figure 12(A-T).** Change in performance measures for various degrees of data drift for synthetically enriched features. For each drift, the left sub-image presents the change in aggregate metrics (AUROC, Precision, Recall, F1-Score, Brier Score), and the right sub-image presents the per-class F1-score. p-values were calculated using a two-sided multivariate maximum mean discrepancy (MMD) statistical test with no correction for multiple comparison. Data are presented as mean values +/- 1 standard deviation.
- **Supplementary Figure 13:** Sampling methodology for synthetic categorical drifts.S1 represents the source dataset which is composed of a random sampling of patients matching the distribution found in the larger dataset. To create the synthetic drift (S2,S3, and S4), we have a percentage of images belonging to a single class added to inflate their representation (represented by red). The remaining images (represented by blue) match a stratified random sample from the general population (i.e., the source dataset, matching the distribution found in S1).

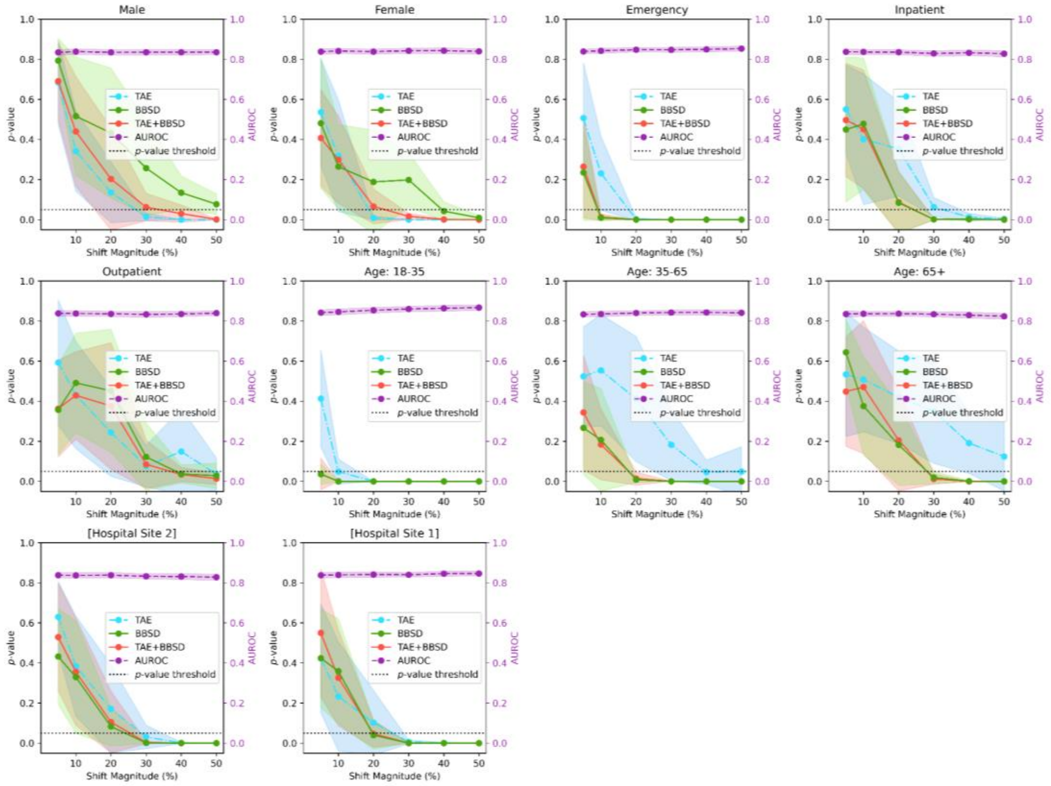

Supplementary Figure 1.

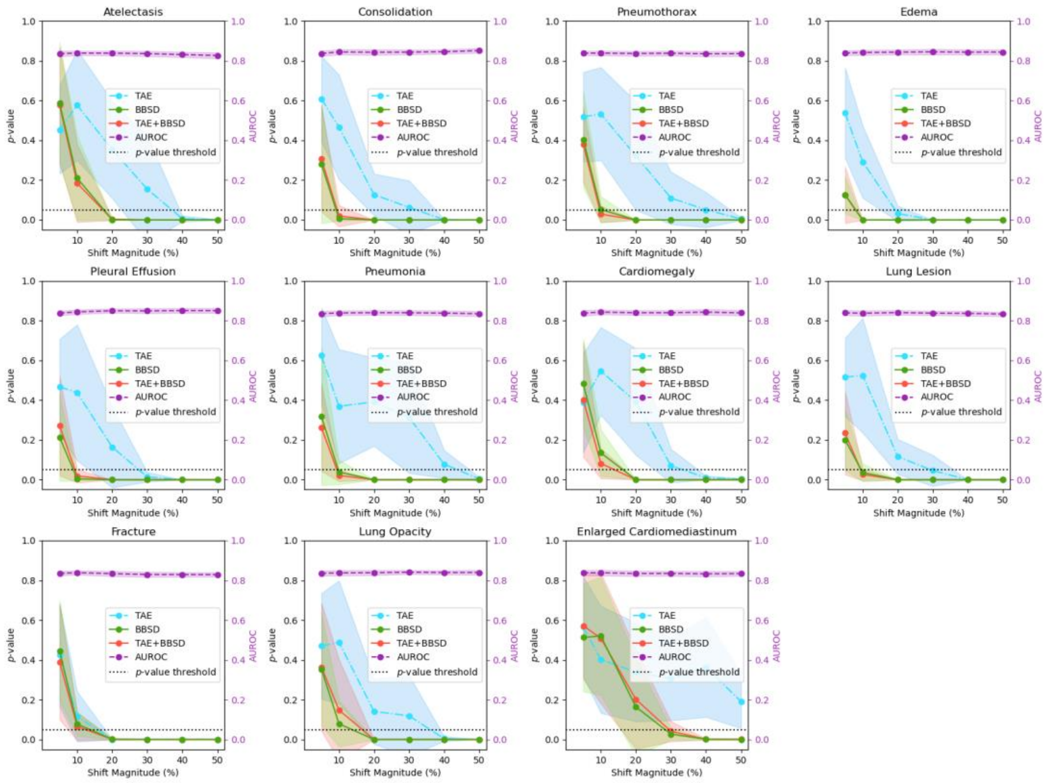

Supplementary Figure 2.

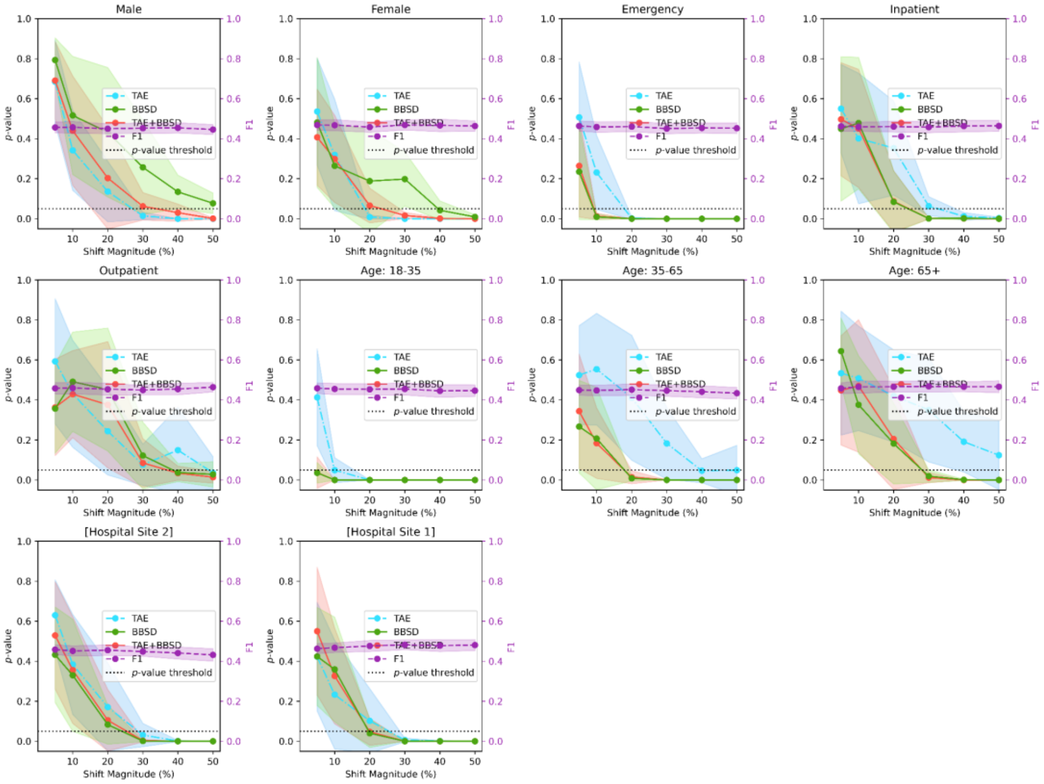

Supplementary Figure 3.

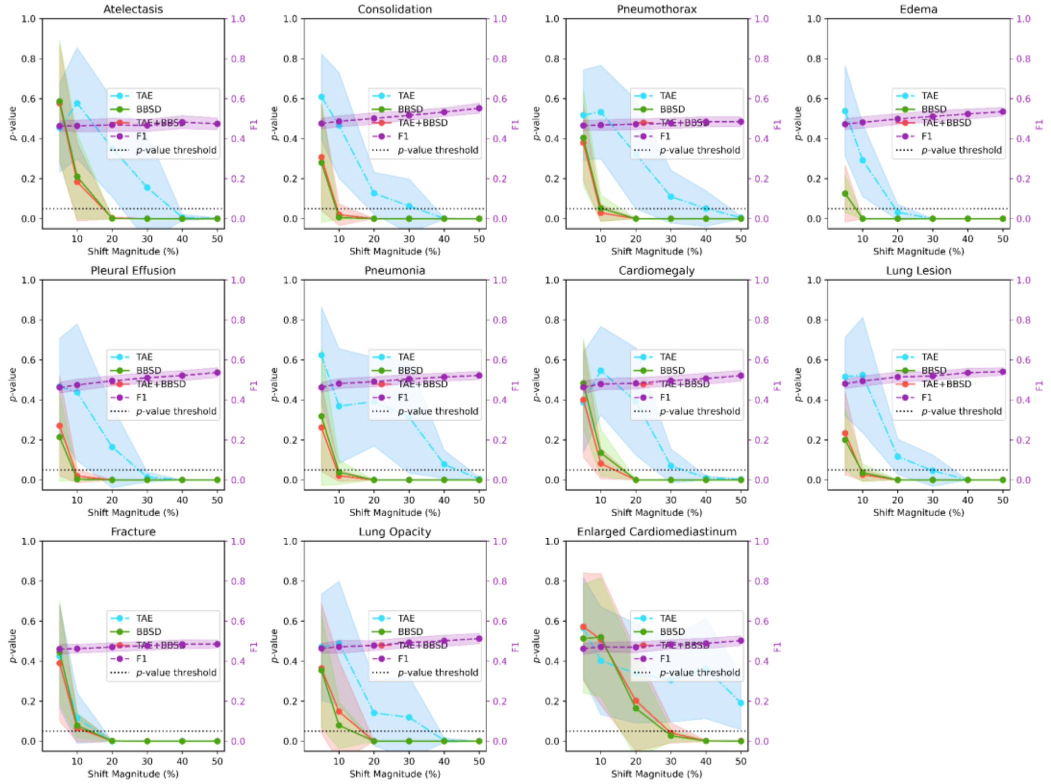

Supplementary Figure 4.

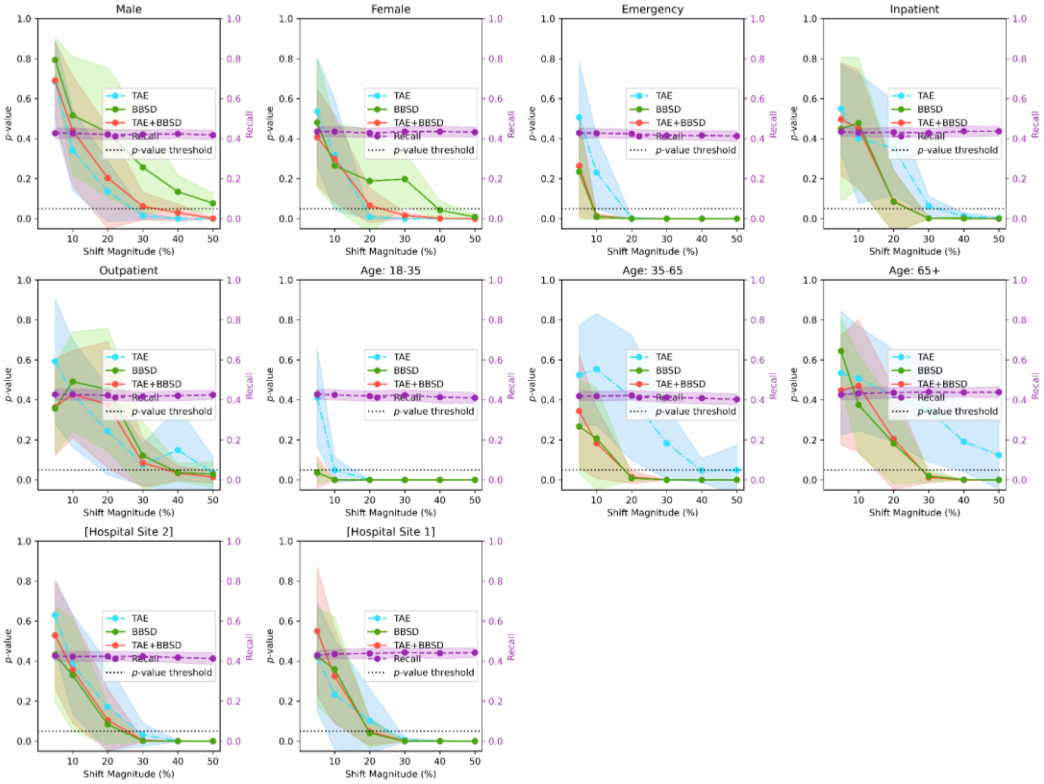

Supplementary Figure 5.

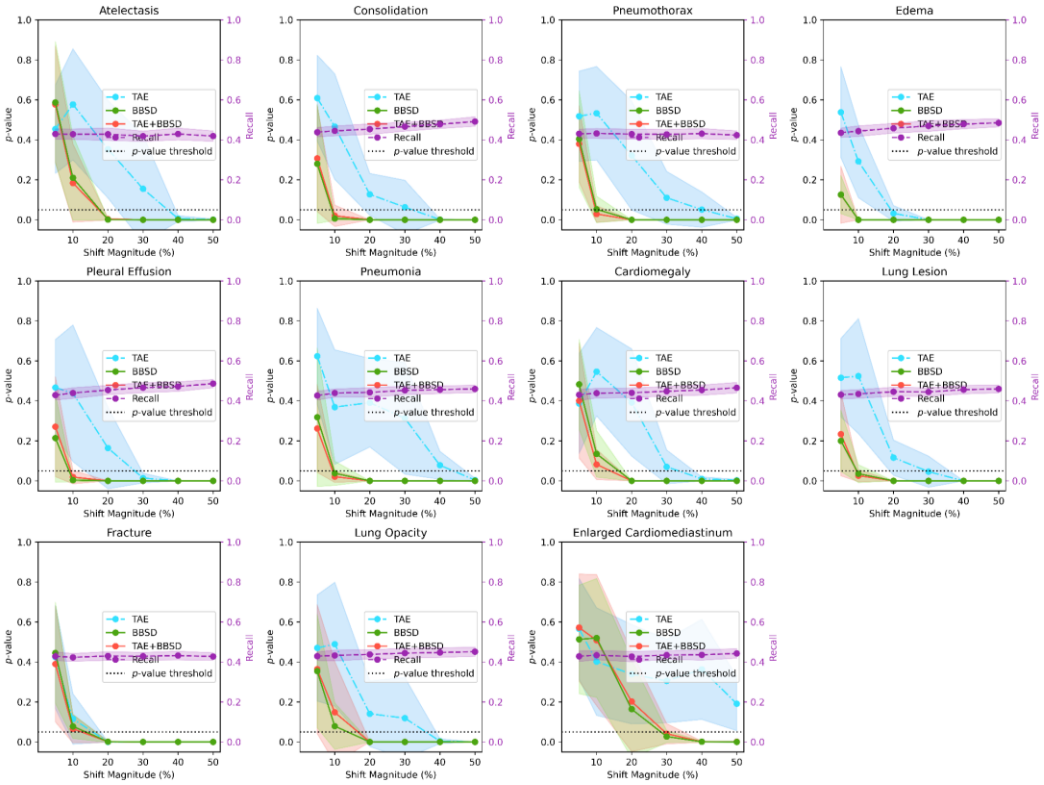

Supplementary Figure 6.

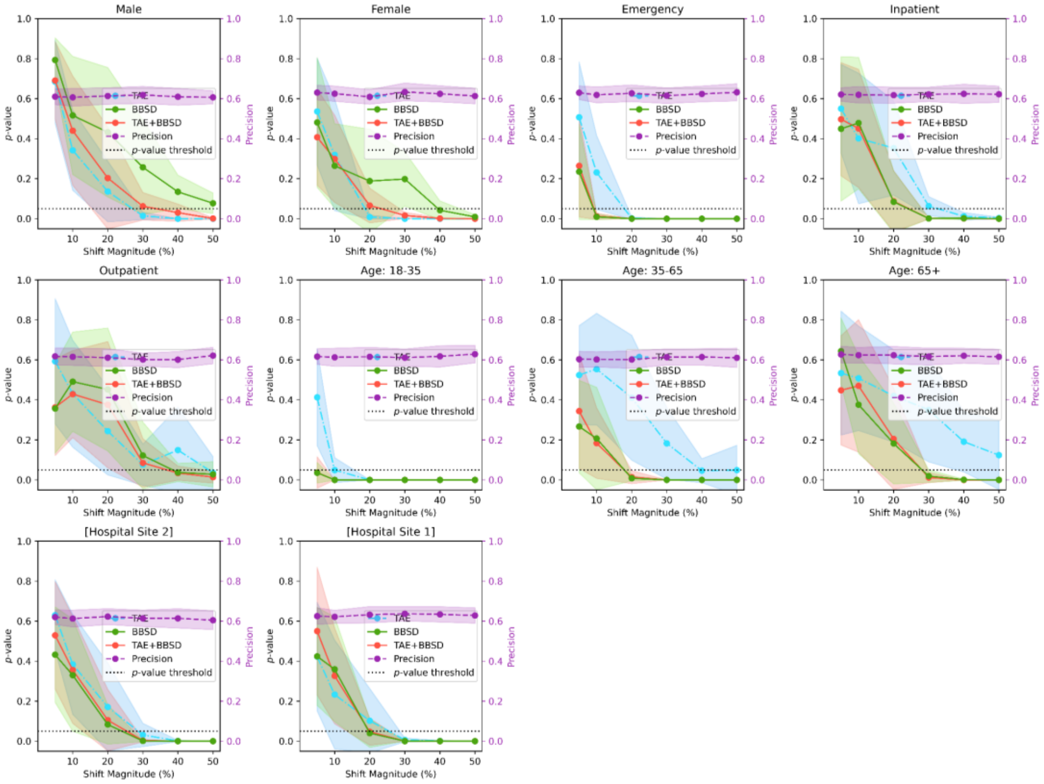

Supplementary Figure 7.

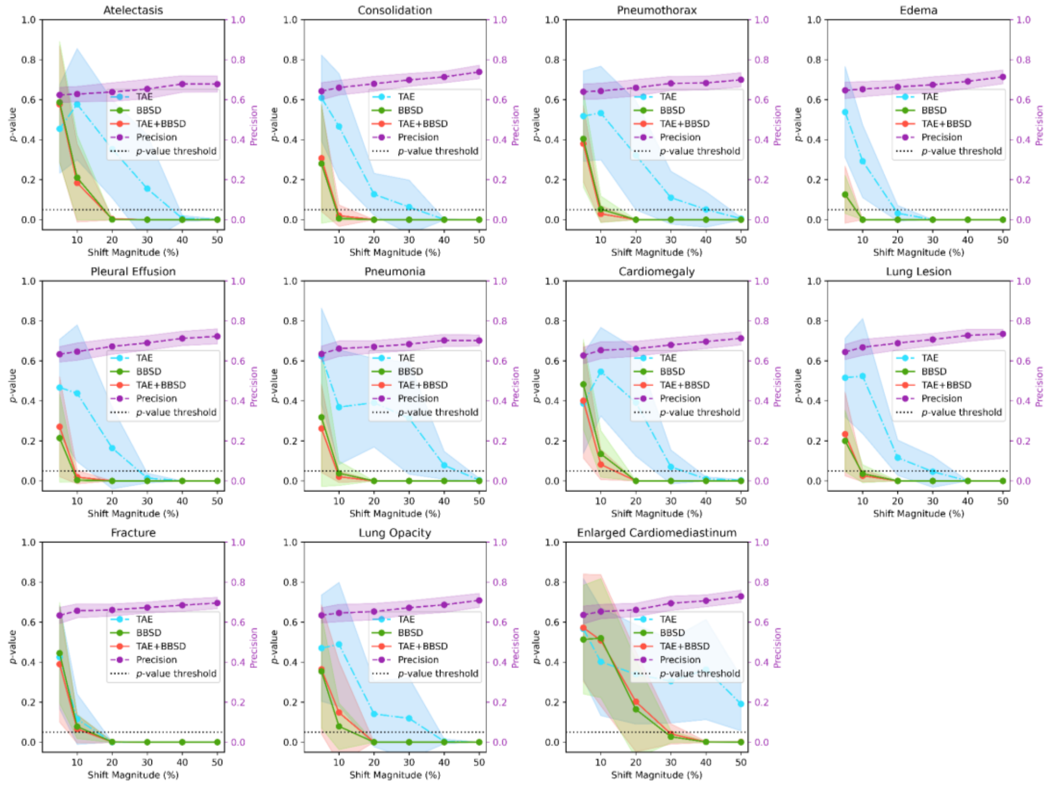

Supplementary Figure 8.

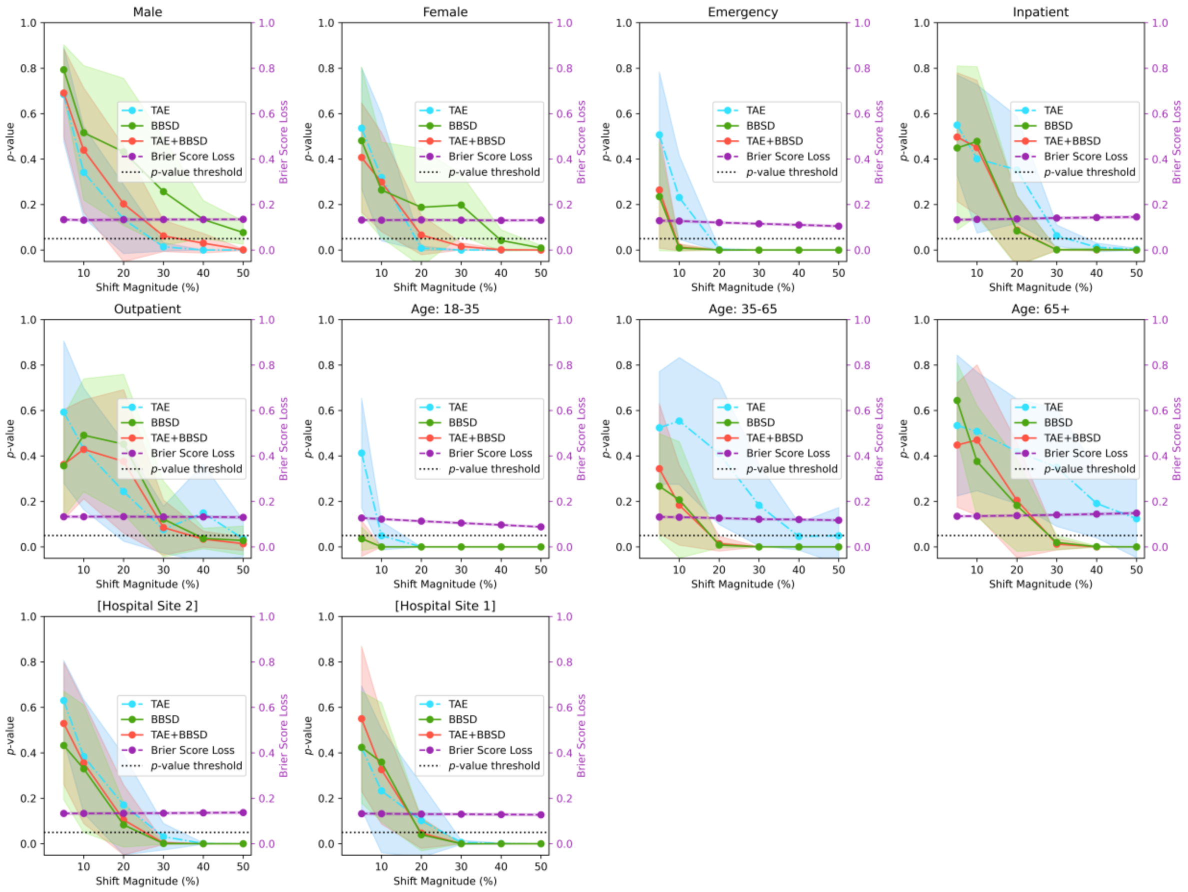

Supplementary Figure 9.

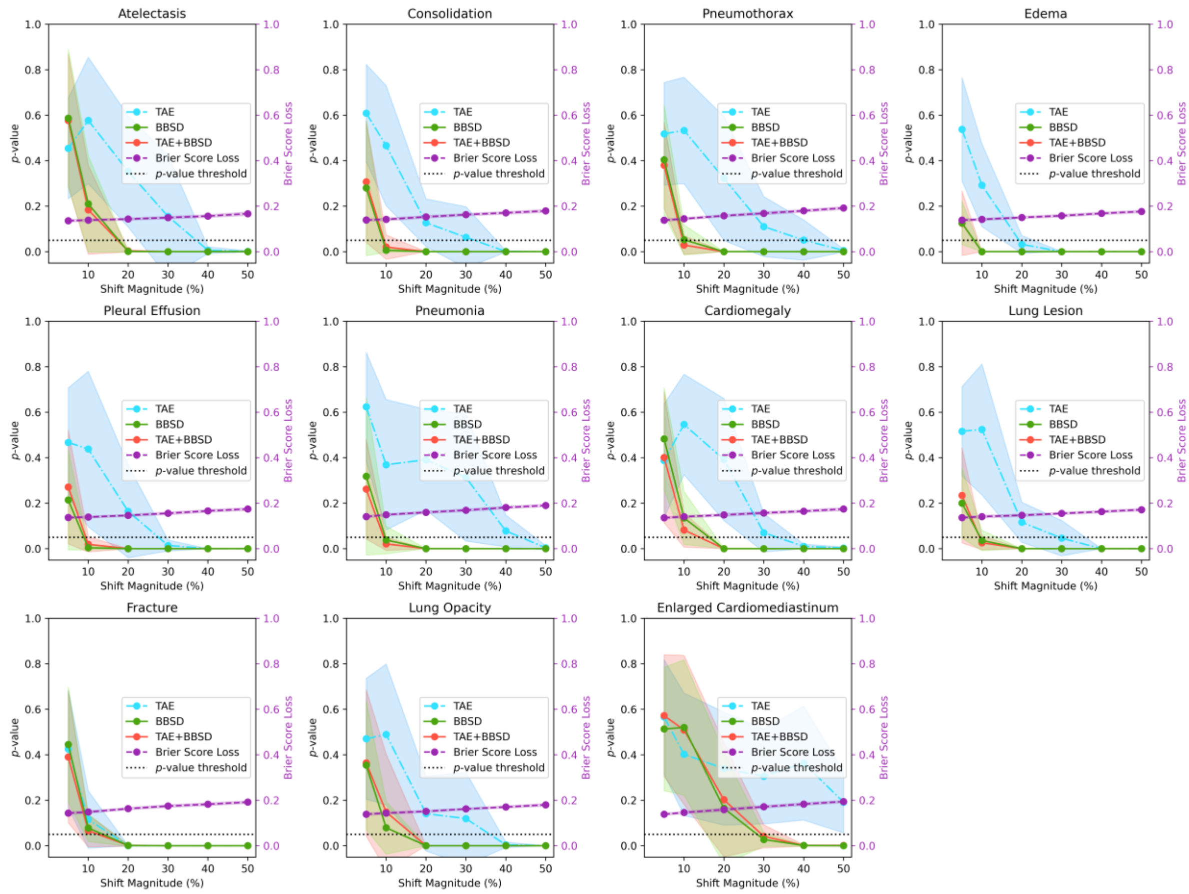

Supplementary Figure 10.

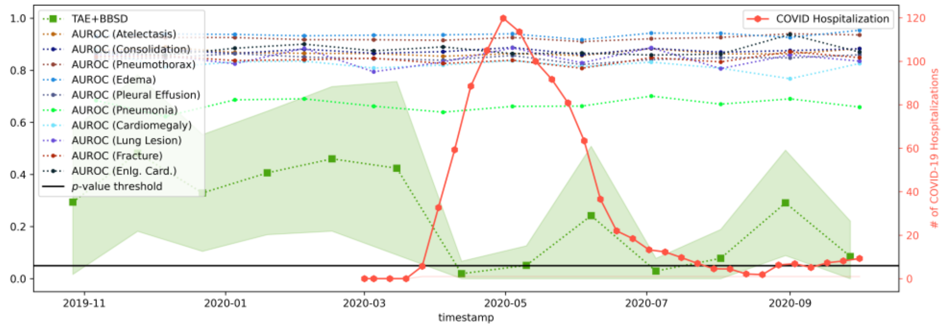
TAE+BBSD
AUROC (Atelectasis)
AUROC (Consolidation)
AUROC (Pneumothorax)
AUROC (Edema)
AUROC (Pleural Effusion)
AUROC (Pneumonia)
AUROC (Cardiomegaly)
AUROC (Lung Lesion)
AUROC (Fracture)
AUROC (Enlg. Card.)
p-value threshold
COVID Hospitalization
timestamp
# of COVID-19 Hospitalizations
2019-11
2020-01
2020-03
2020-05
2020-07
2020-09

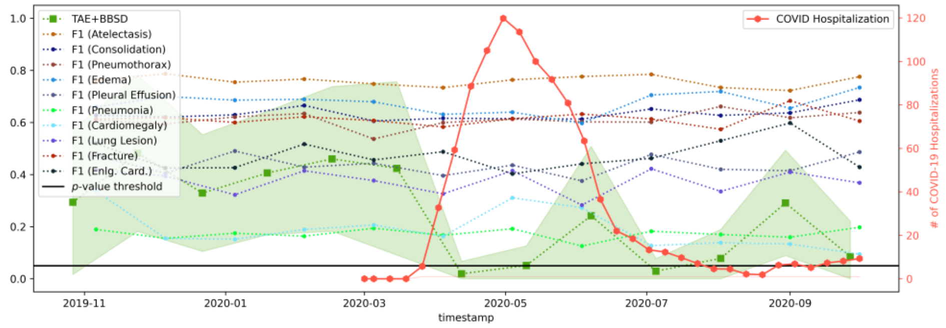
TAE+BBSD
F1 (Atelectasis)
F1 (Consolidation)
F1 (Pneumothorax)
F1 (Edema)
F1 (Pleural Effusion)
F1 (Pneumonia)
F1 (Cardiomegaly)
F1 (Lung Lesion)
F1 (Fracture)
F1 (Enlg. Card.)
p-value threshold
COVID Hospitalization
timestamp
# of COVID-19 Hospitalizations
2019-11
2020-01
2020-03
2020-05
2020-07
2020-09

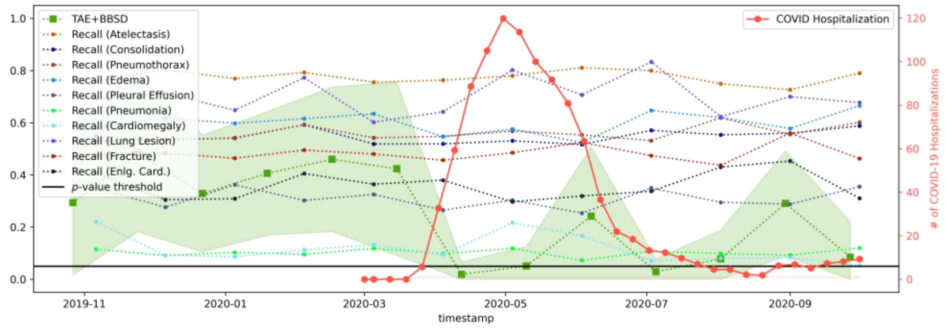
TAE+BBSD
Recall (Atelectasis)
Recall (Consolidation)
Recall (Pneumothorax)
Recall (Edema)
Recall (Pleural Effusion)
Recall (Pneumonia)
Recall (Cardiomegaly)
Recall (Lung Lesion)
Recall (Fracture)
Recall (Enlg. Card.)
p-value threshold
COVID Hospitalization
timestamp
# of COVID-19 Hospitalizations
2019-11
2020-01
2020-03
2020-05
2020-07
2020-09

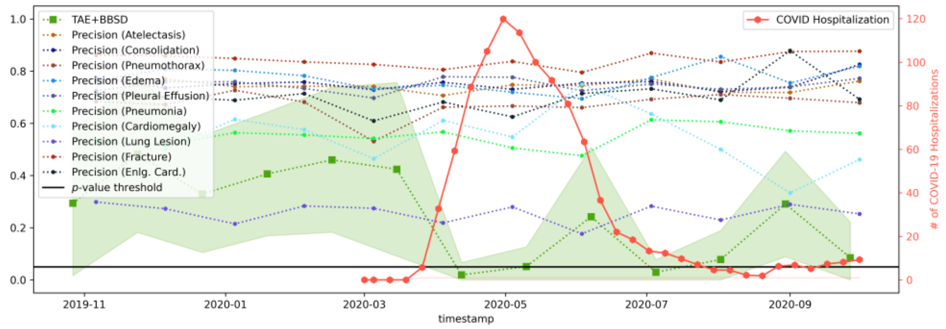
TAE+BBSD
Precision (Atelectasis)
Precision (Consolidation)
Precision (Pneumothorax)
Precision (Edema)
Precision (Pleural Effusion)
Precision (Pneumonia)
Precision (Cardiomegaly)
Precision (Lung Lesion)
Precision (Fracture)
Precision (Enlg. Card.)
p-value threshold
COVID Hospitalization
timestamp
# of COVID-19 Hospitalizations
2019-11
2020-01
2020-03
2020-05
2020-07
2020-09

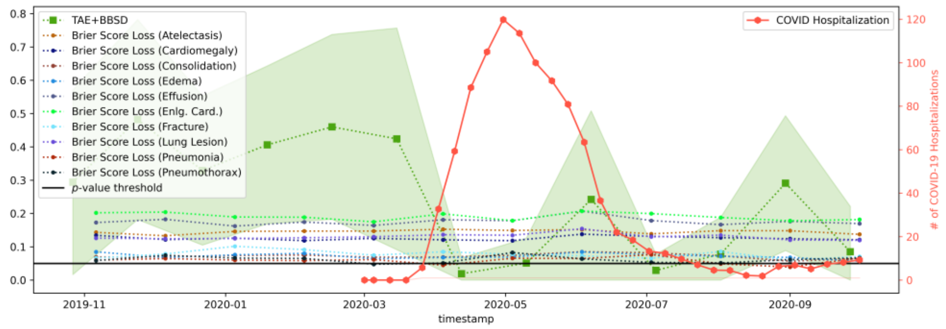
TAE+BBSD
Brier Score Loss (Atelectasis)
Brier Score Loss (Cardiomegaly)
Brier Score Loss (Consolidation)
Brier Score Loss (Edema)
Brier Score Loss (Effusion)
Brier Score Loss (Enlg. Card.)
Brier Score Loss (Fracture)
Brier Score Loss (Lung Lesion)
Brier Score Loss (Pneumonia)
Brier Score Loss (Pneumothorax)
p-value threshold
COVID Hospitalization
timestamp
# of COVID-19 Hospitalizations
2019-11
2020-01
2020-03
2020-05
2020-07
2020-09

Supplementary Figure 11.

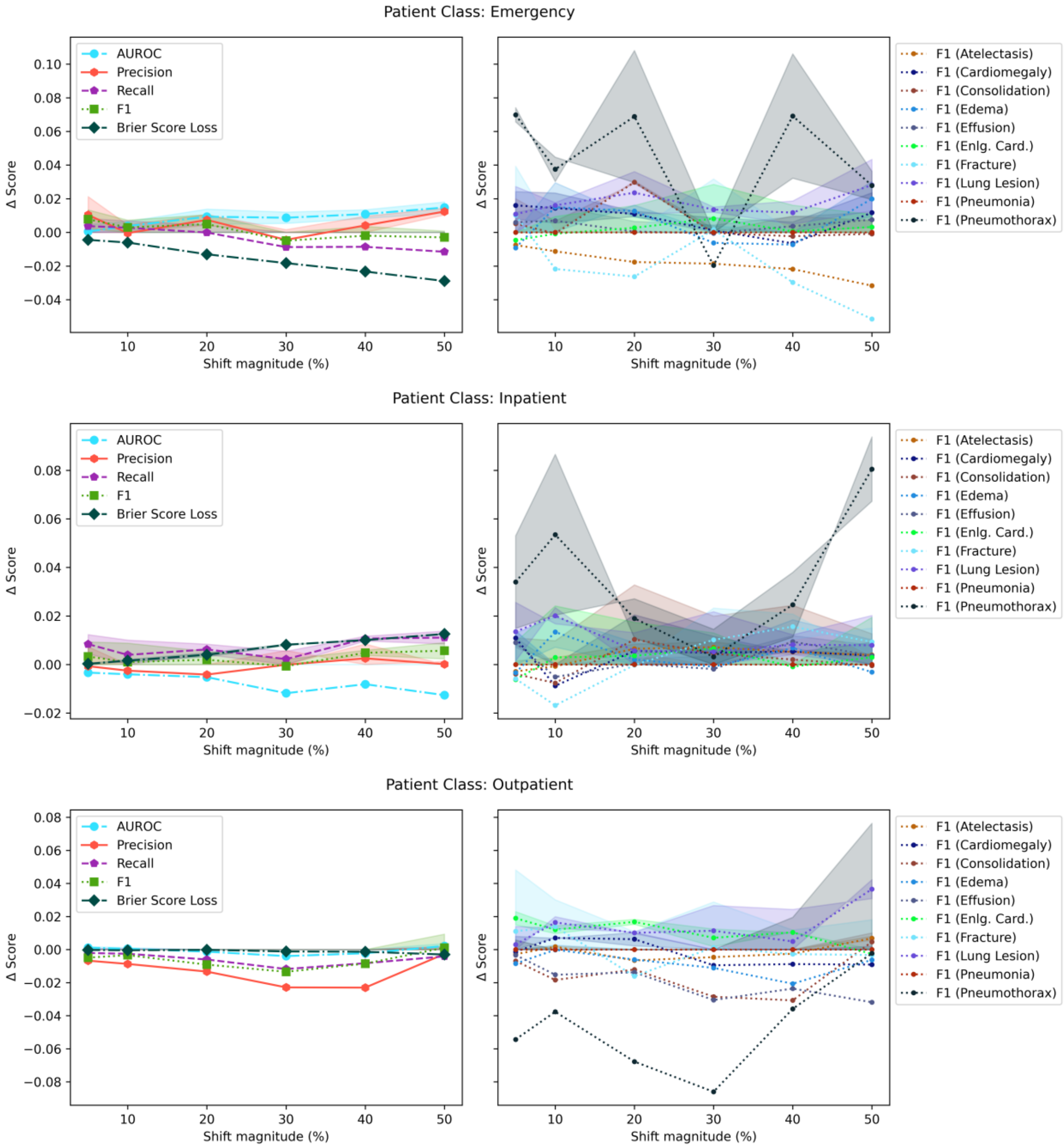

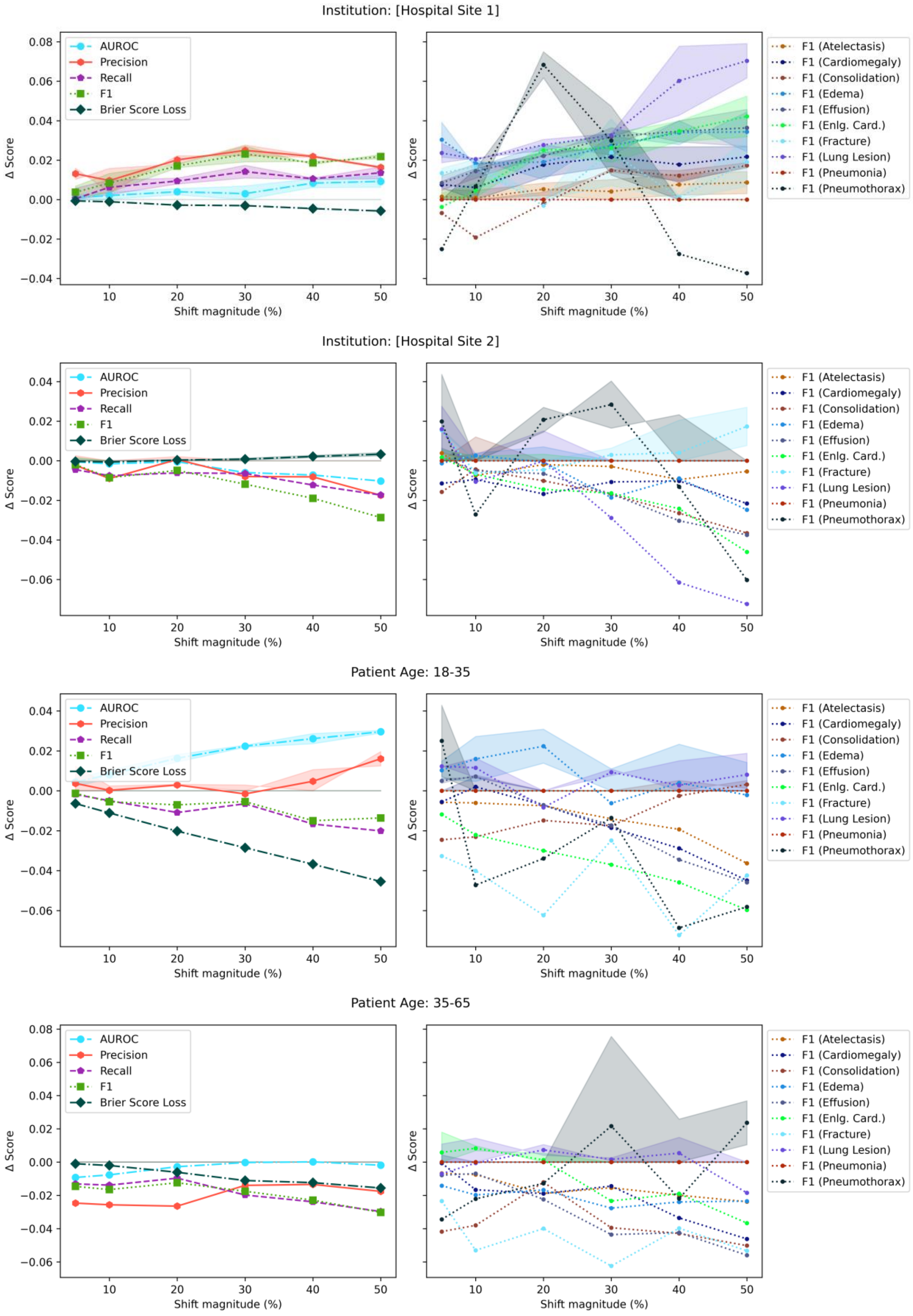
Institution: [Hospital Site 1]
Institution: [Hospital Site 2]
Patient Age: 18-35
Patient Age: 35-65
AUROC
Precision
Recall
F1
Brier Score Loss
F1 (Atelectasis)
F1 (Cardiomegaly)
F1 (Consolidation)
F1 (Edema)
F1 (Effusion)
F1 (Enlg. Card.)
F1 (Fracture)
F1 (Lung Lesion)
F1 (Pneumonia)
F1 (Pneumothorax)
Δ Score
Shift magnitude (%)

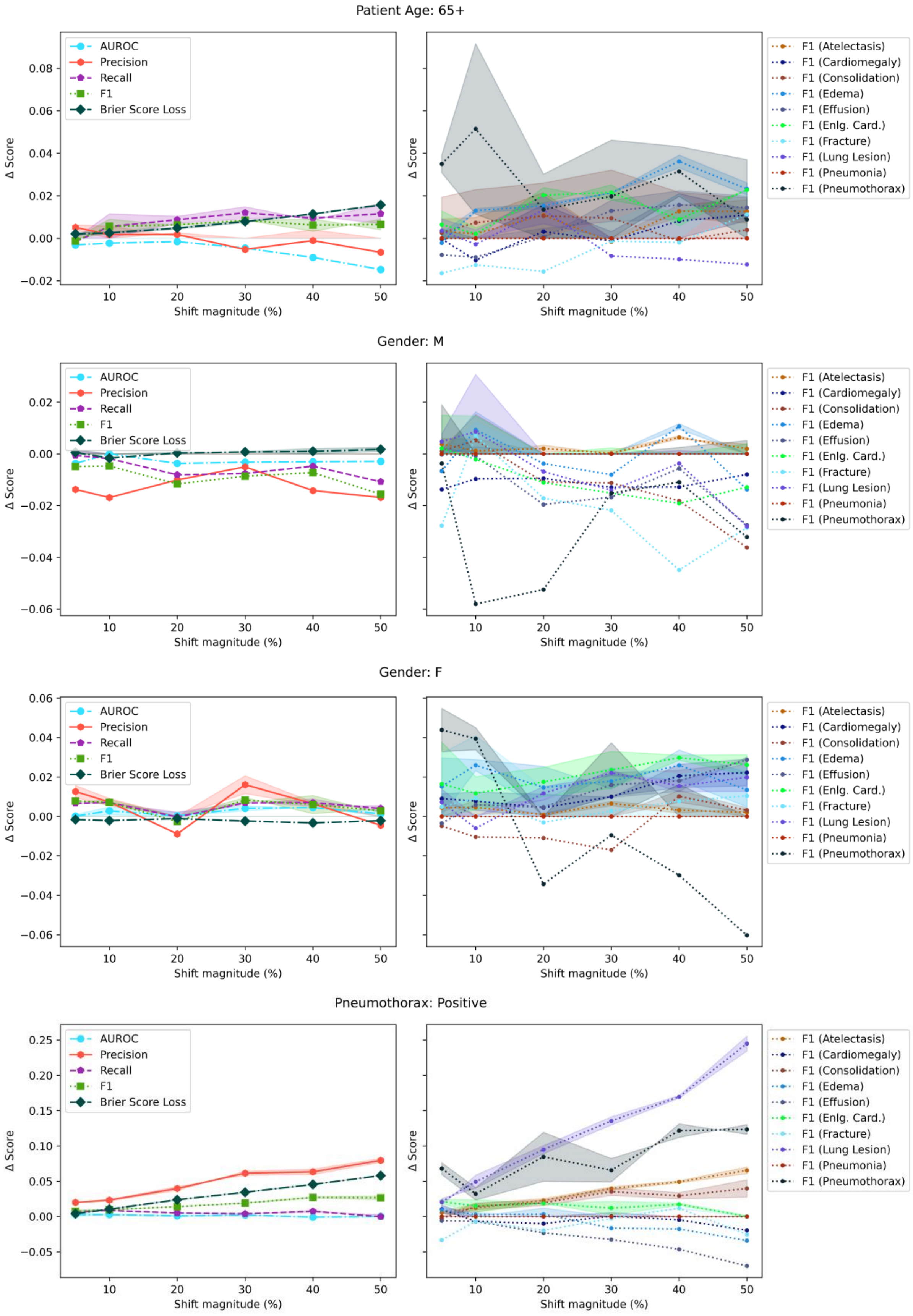

Patient Age: 65+
Gender: M
Gender: F
Pneumothorax: Positive
AUROC
Precision
Recall
F1
Brier Score Loss
F1 (Atelectasis)
F1 (Cardiomegaly)
F1 (Consolidation)
F1 (Edema)
F1 (Effusion)
F1 (Enlg. Card.)
F1 (Fracture)
F1 (Lung Lesion)
F1 (Pneumonia)
F1 (Pneumothorax)
Δ Score
Shift magnitude (%)

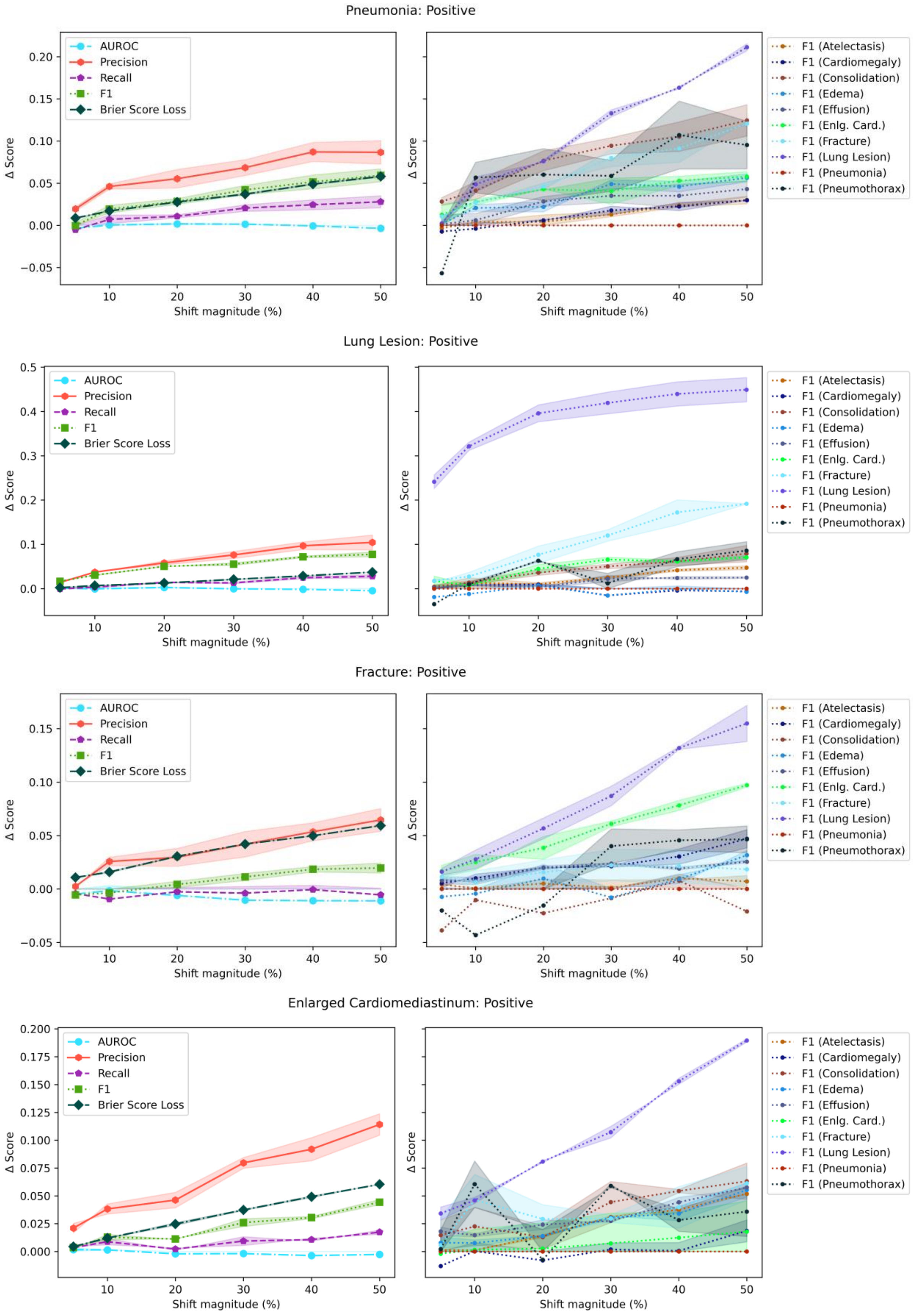
Pneumonia: Positive
Lung Lesion: Positive
Fracture: Positive
Enlarged Cardiomediastinum: Positive
Δ Score
Shift magnitude (%)
AUROC
Precision
Recall
F1
Brier Score Loss
F1 (Atelectasis)
F1 (Cardiomegaly)
F1 (Consolidation)
F1 (Edema)
F1 (Effusion)
F1 (Enlg. Card.)
F1 (Fracture)
F1 (Lung Lesion)
F1 (Pneumonia)
F1 (Pneumothorax)

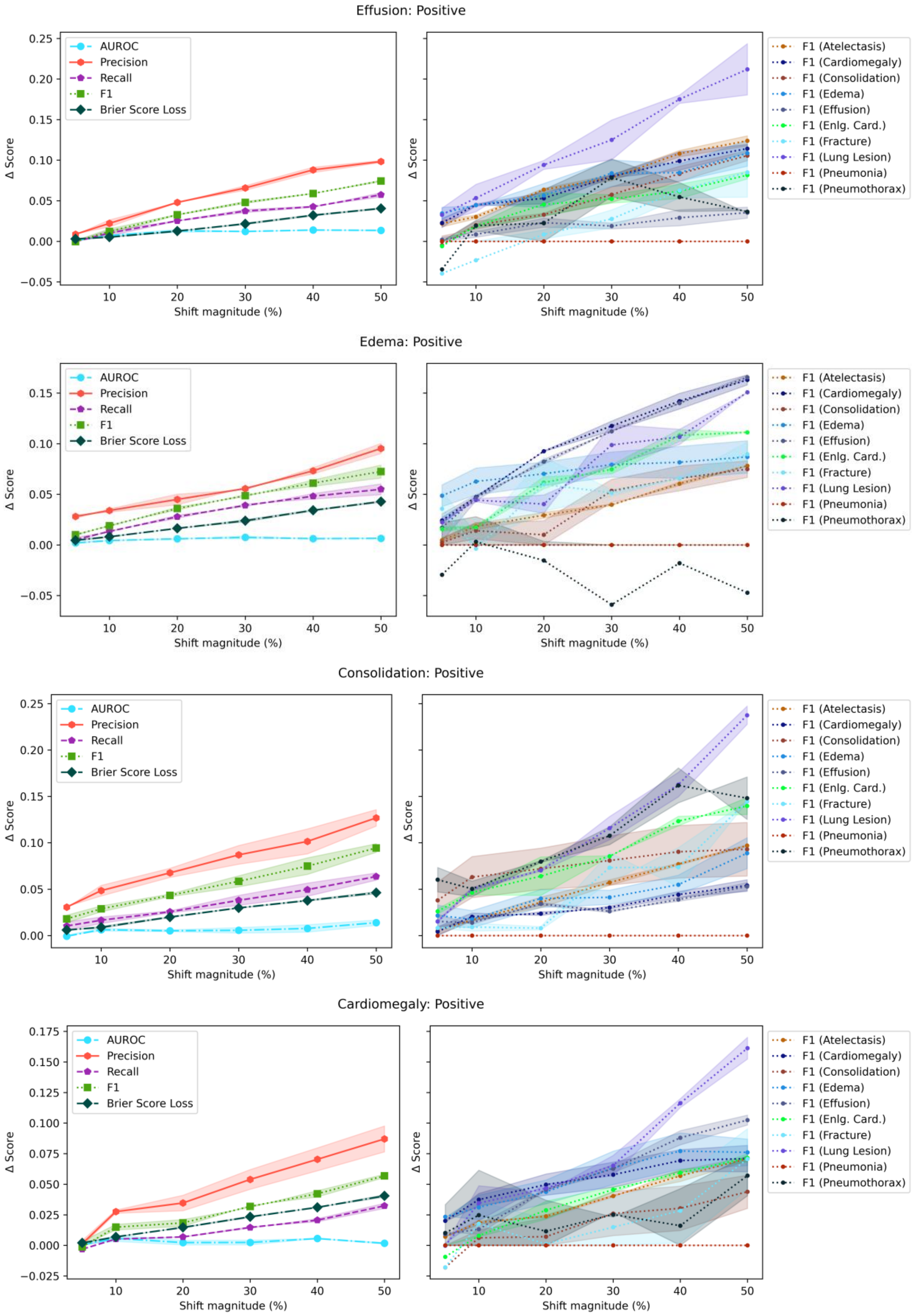
Effusion: Positive
AUROC
Precision
Recall
F1
Brier Score Loss
Δ Score
Shift magnitude (%)
F1 (Atelectasis)
F1 (Cardiomegaly)
F1 (Consolidation)
F1 (Edema)
F1 (Effusion)
F1 (Enlg. Card.)
F1 (Fracture)
F1 (Lung Lesion)
F1 (Pneumonia)
F1 (Pneumothorax)
Edema: Positive
Consolidation: Positive
Cardiomegaly: Positive

Atelectasis: Positive

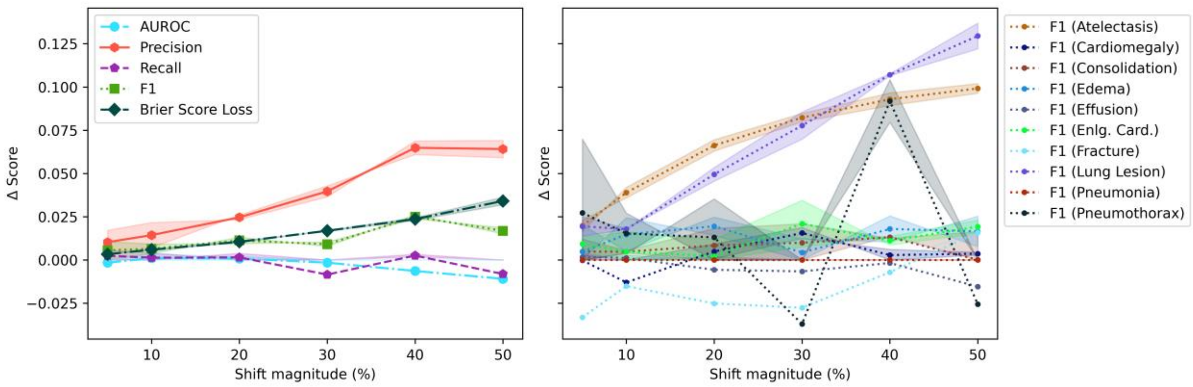

Supplementary Figure 12(A-T).

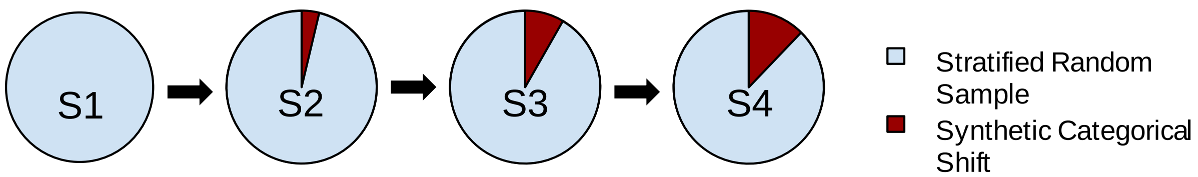

Supplementary Figure 13.
