# Supplementary material for: Empirical data drift detection experiments on real-world medical imaging data
Source: Nat Commun. 2024 Feb 29;15:1887. doi: 10.1038/s41467-024-46142-w (PMC10904813; doi:10.1038/s41467-024-46142-w)
Supplement: Supplementary file 3 — Reporting Summary [file 41467_2024_46142_MOESM3_ESM.pdf]

nature portfolio

Corresponding author(s): Mohamed Abdalla

Last updated by author(s): Jan 23, 2024

Reporting Summary

Nature Portfolio wishes to improve the reproducibility of the work that we publish. This form provides structure for consistency and transparency in reporting. For further information on Nature Portfolio policies, see our Editorial Policies and the Editorial Policy Checklist.

Statistics

For all statistical analyses, confirm that the following items are present in the figure legend, table legend, main text, or Methods section.

n/a	Confirmed	
☐	☒	The exact sample size (*n*) for each experimental group/condition, given as a discrete number and unit of measurement
☐	☒	A statement on whether measurements were taken from distinct samples or whether the same sample was measured repeatedly
☐	☒	The statistical test(s) used AND whether they are one- or two-sided<br>*Only common tests should be described solely by name; describe more complex techniques in the Methods section.*
☐	☒	A description of all covariates tested
☐	☒	A description of any assumptions or corrections, such as tests of normality and adjustment for multiple comparisons
☐	☒	A full description of the statistical parameters including central tendency (e.g. means) or other basic estimates (e.g. regression coefficient) AND variation (e.g. standard deviation) or associated estimates of uncertainty (e.g. confidence intervals)
☐	☒	For null hypothesis testing, the test statistic (e.g. *F*, *t*, *r*) with confidence intervals, effect sizes, degrees of freedom and *P* value noted<br>*Give P values as exact values whenever suitable.*
☒	☐	For Bayesian analysis, information on the choice of priors and Markov chain Monte Carlo settings
☒	☐	For hierarchical and complex designs, identification of the appropriate level for tests and full reporting of outcomes
☒	☐	Estimates of effect sizes (e.g. Cohen's *d*, Pearson's *r*), indicating how they were calculated

Our web collection on statistics for biologists contains articles on many of the points above.

Software and code

Policy information about availability of computer code

Data collection	Data was collected from hospital databases using custom SQL scripts.
Data analysis	We used custom scripts (Python 3.9) to perform the data analysis. Code for our experiments and analysis is being made public.

For manuscripts utilizing custom algorithms or software that are central to the research but not yet described in published literature, software must be made available to editors and reviewers. We strongly encourage code deposition in a community repository (e.g. GitHub). See the Nature Portfolio guidelines for submitting code & software for further information.

Data

Policy information about availability of data

All manuscripts must include a data availability statement. This statement should provide the following information, where applicable:

- Accession codes, unique identifiers, or web links for publicly available datasets
- A description of any restrictions on data availability
- For clinical datasets or third party data, please ensure that the statement adheres to our policy

The dataset from this study is held securely in coded form at the Institute for Better Health, Trillium Health Partners and is not openly available due to privacy concerns and legal requirements. However, access may be granted to those who meet criteria for confidential access. Please contact Dr. Mohamed Abdalla or Dr. Benjamin Fine at first.last@thp.ca .

Research involving human participants, their data, or biological material

Policy information about studies with human participants or human data. See also policy information about sex, gender (identity/presentation), and sexual orientation and race, ethnicity and racism.

Reporting on sex and gender	We did sub-analysis to study the effect of sex on our findings. Our institution does not collect gender information, so we could not perform this sub-analysis.
Reporting on race, ethnicity, or other socially relevant groupings	Trillium Health Partners does not collect race or ethnicity data. As such, these groupings, while important, were not included in our analysis.
Population characteristics	All patients over the age of 18 who received chest radiographs at any Trillium Health Partner hospital over the specified study dates (January 2016 and December 2020) were included into the study population. This time-frame covers the introduction of COVID-19 into our population. All patients were included (there were no other exclusion criteria). Supplementary Table 1 provides descriptive statistics of our study population.
Recruitment	This was a retrospective study on all patients who received chest radiographs between two dates. There was no active recruitment of patients.
Ethics oversight	The protocol was approved by Trillium Health Partner's research ethics board (Protocol ID: #1031).

Note that full information on the approval of the study protocol must also be provided in the manuscript.

Field-specific reporting

Please select the one below that is the best fit for your research. If you are not sure, read the appropriate sections before making your selection.

☒ Life sciences ☐ Behavioural & social sciences ☐ Ecological, evolutionary & environmental sciences

For a reference copy of the document with all sections, see nature.com/documents/nr-reporting-summary-flat.pdf

Life sciences study design

All studies must disclose on these points even when the disclosure is negative.

Sample size	No sample size calculations were done, as one of our contributions was studying the effect of sample size on drift detection. Our study population size was over 200,000 which is amongst the largest used for drift detection experiments.
Data exclusions	We excluded patients non-adult patients.
Replication	Each experiment was run multiple times (with different random sampling of patients) to ensure that the results could be replicated. The entire set of experiments was run twice (to satisfy reviewer requests with the addition of a new metric). The results remained the same. The code of our study will be made fully available online upon publication.
Randomization	Allocation was random for the original sample. For samples that were artificially enriched, the added samples were randomly selected from studies which fit the enrichment criteria. This is discussed in the Methods section of the paper.
Blinding	Blinding was not relevant to the study as we were not comparing an effect between two groups.

Reporting for specific materials, systems and methods

We require information from authors about some types of materials, experimental systems and methods used in many studies. Here, indicate whether each material, system or method listed is relevant to your study. If you are not sure if a list item applies to your research, read the appropriate section before selecting a response.

Materials & experimental systems

n/a	Involved in the study	
☒	☐	Antibodies
☒	☐	Eukaryotic cell lines
☒	☐	Palaeontology and archaeology
☒	☐	Animals and other organisms
☐	☒	Clinical data
☒	☐	Dual use research of concern
☒	☐	Plants

Methods

n/a	Involved in the study	
☒	☐	ChIP-seq
☒	☐	Flow cytometry
☒	☐	MRI-based neuroimaging

Clinical data

Policy information about clinical studies

All manuscripts should comply with the ICMJE guidelines for publication of clinical research and a completed CONSORT checklist must be included with all submissions.

Clinical trial registration	This was not a clinical trial (hence no CONSORT).
Study protocol	This was not a clinical trial, so pre-registration and other considerations for study protocols did not apply to our study setting.
Data collection	Our experiments use a dataset of 239,235 chest radiographs (CXR) and associated examination reports and meta-data gathered from 78,542 patients over the age of 18 who received imaging at [institution name redacted], a hospital system based in [institution details redacted] between January 2016 and December 2020.
Outcomes	Primary outcome was the sensitivity of drift detection methods.

Plants

Seed stocks	*Report on the source of all seed stocks or other plant material used. If applicable, state the seed stock centre and catalogue number. If plant specimens were collected from the field, describe the collection location, date and sampling procedures.*
Novel plant genotypes	*Describe the methods by which all novel plant genotypes were produced. This includes those generated by transgenic approaches, gene editing, chemical/radiation-based mutagenesis and hybridization. For transgenic lines, describe the transformation method, the number of independent lines analyzed and the generation upon which experiments were performed. For gene-edited lines, describe the editor used, the endogenous sequence targeted for editing, the targeting guide RNA sequence (if applicable) and how the editor was applied.*
Authentication	*Describe any authentication procedures for each seed stock used or novel genotype generated. Describe any experiments used to assess the effect of a mutation and, where applicable, how potential secondary effects (e.g. second site T-DNA insertions, mosiacism, off-target gene editing) were examined.*
